# Pasteurella multocida Bacteremia Secondary to Peritoneal Dialysis Associated Peritonitis: A Case Report and Literature Review

**DOI:** 10.7759/cureus.24188

**Published:** 2022-04-16

**Authors:** John M Giacona, Maxwell Weiner, John Hanna, Tomasz Jodlowski, Roger Bedimo

**Affiliations:** 1 Internal Medicine, Cardiology, University of Texas Southwestern Medical Center, Dallas, USA; 2 School of Health Professions, University of Texas Southwestern Medical Center, Dallas, USA; 3 Division of Infectious Diseases and Geographic Medicine, University of Texas Southwestern Medical Center, Dallas, USA; 4 Infectious Disease, Veterans Affairs North Texas Healthcare System, Dallas, USA

**Keywords:** zoonotic infection, sepsis, antibiotic therapy, bacteremia, peritoneal dialysis, end stage renal disease, pasteurella multocida, peritonitis

## Abstract

We report the fiftieth case in the literature of *Pasteurella* species peritoneal dialysis (PD)-related peritonitis and the third reported case of *Pasteurella multocida* bacteremia associated with PD-related peritonitis. Our review provides the most up-to-date collection of all fifty reported cases of PD-related peritonitis caused by *Pasteurella* species. A 77-year-old Caucasian male with a past medical history significant for new-onset left-ventricular systolic heart failure, severe mitral valve regurgitation, and end-stage renal disease on PD for six months presented to the emergency department with a one-week cloudy peritoneal effluent and intermittent abdominal pain. *Pasteurella multocida *was isolated from blood cultures and peritoneal fluid cultures. The patient was treated with intravenous piperacillin-tazobactam and intraperitoneal cefepime. The PD catheter was not removed or exchanged. A repeat blood culture on the third hospital day was negative. His hospital course was complicated by cardiogenic shock, atrial fibrillation, and gastrointestinal bleeding, and his goals of care changed to focus on comfort measures. This case report and literature review provide a resource for healthcare providers who may encounter this infection in the future. This case also serves as a reminder of the challenges of PD in patients at risk of acquired zoonotic infections from their pets. Based on the reviewed three cases of *Pasteurella multocida* bacteremia associated with PD-related peritonitis, blood cultures may be a prudent option for patients presenting with peritoneal dialysis associated peritonitis to ensure that concurrent bacteremia is not overlooked.

## Introduction

*Pasteurella multocida* (*P. multocida*) is a non-motile coccobacillus of the family *Pasteurellaceae*, first isolated in 1880 by Louis Pasteur [1]. This pathogen is a commensal of the respiratory tract and the oral cavity in 70-90% of cats and 66% of dogs [2]. The first human infection was reported in 1914. *P. multocida* is classically known to cause systemic infections following a cat bite. However, there is a subset of individuals who have experienced peritoneal dialysis (PD)-related peritonitis from *P. multocida*, most associated with having cats at home. Upon literature review, there are 49 reported cases of *Pasteurella* species peritonitis associated with PD catheter. Out of these 49 cases, 47 cases were caused by *P. multocida* [2], one case was caused by* Pasteurella pneumotropica* [3], and another was caused by *Pasteurella dagmatis* [4]. Bacteremia is reported to be a rare complication of PD-related peritonitis, and blood cultures are often not done in the absence of fever or suspected sepsis [5]. There are only two previously reported cases of *P. multocida* bacteremia associated with peritonitis [6,7]. We report the third case of *P. multocida* peritonitis in a 77-year-old patient who presented with cloudy peritoneal effluent and abdominal pain around the PD catheter insertion site, and *P. multocida *was isolated from both the blood cultures and the peritoneal fluid culture.

## Case presentation

The patient is a 77-year-old Caucasian male with a past medical history significant for end-stage renal disease (ESRD) who has been on chronic ambulatory PD for six months. The patient presented with intermittent peri-peritoneal dialysis catheter site pain that progressed to generalized abdominal pain. During his last dialysis session prior to the presentation, he noted that the dialysis effluent was cloudy, and he was referred to the emergency department. He did not have fever, chills, or night sweats.

His past medical history included type-2 diabetes mellitus, hypertension, coronary artery disease status after 3-vessel coronary artery bypass graft and percutaneous coronary intervention with drug-eluting stent placement, peripheral arterial disease post arterial bypass, recently diagnosed (before presentation's symptoms onset) new-onset systolic heart failure, severe mitral regurgitation, benign prostate hypertrophy, and hypothyroidism. He had no previous episodes of peritonitis. He has a cat at home and reported that the cat previously bit him when he was undergoing peritoneal dialysis.

At presentation, he was afebrile (96.8 degrees Fahrenheit), with a blood pressure of 85/39 mmHg, heart rate of 96, respiratory rate of 22, and oxygen saturation of 98% on room air. On physical exam, the patient was in no acute distress. Cardio-pulmonary examination revealed normal and clear breath sounds bilaterally with a murmur consistent with mitral valve regurgitation. His abdomen was soft and non-distended, and the peritoneal dialysis catheter was located in the hypogastric region with no surrounding erythema or warmth but mild tenderness. There were normoactive bowel sounds on auscultation in all four quadrants. The rest of his physical exam was unremarkable.

Initial laboratory tests are summarized in Table 1. A computed tomography scan of his abdomen found no evidence of perforation, inflammation, fluid collections, or abscesses.

**Table 1:** Pertinent laboratory results on presentation

Laboratory test	Result
White blood cell (WBC) count	12.1 k/uL
Neutrophil count	9.67 K/uL
Hemoglobin	14.9 g/dL
Platelet count	284 k/uL
Sodium	134 mEq/L
Potassium	2.8 mEq/L
Anion gap	20
Lactic acid	3.7 mmol/L
Brain natriuretic peptide	>4900
Troponin I	0.2 ng/ml
Hemoglobin A1C	6.70%
Peritoneal fluid appearance	Cloudy
Peritoneal fluid WBC count	994/ uL

He empirically received one dose of vancomycin 1.25 grams and started on piperacillin-tazobactam 2.25 mg every eight hours. Blood cultures and peritoneal cultures were obtained prior to initiation of the antimicrobial therapy and returned positive the next day for gram-negative rods that subsequently were identified as *P. multocida*. The patient was continued on piperacillin-tazobactam 2.25 mg every 8eight hours and was additionally given intraperitoneal cefepime 1 gram at a four-hour dwell daily while awaiting blood culture susceptibilities results that were sent out to a reference lab. On the third and fifth days after presentation, repeat blood cultures were negative. On the fourth and eighth days after presentation, repeat peritoneal fluid cultured were negative. Peritoneal fluid white blood cell (WBC) count also declined to 19uL on the fourth day and 10/uL on the eighth day compared to the presentation with an initial count of 994/uL. The patient received a total of 10 days of intravenous piperacillin-tazobactam and 12 days of intraperitoneal cefepime that was optioned by nephrology to be continued post his peritoneal dialysis sessions. Ten days after presentation, *P. multocida* culture susceptibility results returned susceptible to penicillin (minimum inhibitory concentration [MIC] of 0.5mcg/mL) and ceftriaxone (MIC of ≤0.12 mcg/mL).

On the fourth day of hospitalization, after the patient's initial clinical improvement, the patient underwent elective left-side and right-sided heart catheterization to evaluate for cardiomyopathy. He was found to be in cardiogenic shock with a cardiac index of 1.4, and Swan-Ganz was left in place post the procedure, which required his transfer to the cardiac intensive care unit. His hospital course was further complicated by gastrointestinal bleeding while he was on heparin infusion and dual anti-platelet therapy. Over the following few days of hospitalization, the patient encountered a rapid decline in his overall health; the patient was transitioned to hospice care based on goals of care discussion and his poor overall prognosis.

## Discussion

*P. multocida* causes diverse infectious processes in humans, such as cellulitis, pulmonary infections, meningitis, septic arthritis, and osteomyelitis. Bacteremia associated with PD-related peritonitis secondary to *P. multocida *is rare. The most attributable exposure in 90% of the previously reported* P. multocida* peritonitis cases is related to having cats at home. The transmission was previously reported to occur through licks, bites, and scratches by cats. Additionally, previous reports described contamination of the dialysis machine and tubes by the pets' oropharyngeal organism and colonization of the patients' oropharynx by *P. multocida* [1-50].

Bacteremia is a rare complication of PD-related peritonitis (Table 2) [1-50]. Of the two cases where the *P. multocida *peritonitis was complicated by bacteremia, the first case discussed a patient who presented in an immunocompromised state secondary to systemic lupus erythematosus steroid treatment. This patient suffered peritonitis and bacteremia that progressed to sepsis with vascular collapse, distal ischemia, and respiratory failure [6]. The second case was in an immunocompetent patient who had bacteremia with no signs of sepsis or circulatory shock [7]. Our patient was a host with significant cardio-pulmonary comorbidities and well-controlled diabetes mellitus.

**Table 2:** Reported cases of Pasteurella sp. peritoneal dialysis associated peritonitis IV - intravenous; IP - intraperitoneal; PO - oral

Case No.	Author(s)	Year of publication	Age, gender	Blood culture reported?	Peritoneal culture organism	Animal exposure	Empiric Antibiotics	Culture-driven antibiotics	Peritoneal catheter removed?	Comments
1	Paul and Rostand [18]	1987	55, female	Yes, negative	P. multocida	Cat	Vancomycin (IV) and gentamicin (IV)	Gentamicin (IV)	-	Cat punctured dialysis tubing
2	Elsey et al. [19]	1991	25, male	No	P. multocida	Cat	Cephradine (IP) and gentamicin (IP)	Cephradine (IP) and gentamicin (IP)	-	Cat sleeps with him during dialysis treatments
3	Frankel and Cassidy [20]	1991	55, male	No	P. multocida	Cat	Vancomycin (IP) and gentamicin (IP)	Gentamicin (IP) and ciprofloxacin (PO)	-	Cat plays with him during dialysis treatments
4	London and Bottone [21]	1991	54, male	No	P. multocida	Cat	Vancomycin (IV) and gentamicin (IV)	Cefazolin (PO)	-	Cat punctured dialysis tubing
5	Kitching et al. [22]	1996	75, male	No	P. multocida	Cat	Vancomycin (IV)	Cefamandole (IV)	-	Tubing punctured with claw from cat
6	Uribarri et al. [23]	1996	42, female	No	P. multocida	Cat and dog	Vancomycin (IV)	Gentamicin (IP) and penicillin (PO)	-	Cat bite most likely, assumed from history
7	Loghman [24]	1997	12, female	No	P. multocida	Cat	Cephapirin (IP)	Gentamicin (IP)	-	Puncture of dialysis tubing from cat
8	Mackay, Brown and Hudson [25]	1997	73, male	No	P. multocida	Cat	Vancomycin (IP) and ceftazidime (IP)	Ceftazidime	-	Denies cat nearby during treatments
9	Joh et al. [26]	1998	55, male	Yes, negative	P. multocida	Cat	Vancomycin (IP), gentamicin (IV) and gent (IP)	Ampicillin-sulbactam (PO)	-	Cat playing with dialysis tubing
10	Musio and Tiu [27]	1998	46, female	No	P. multocida	Cat	Pipracillin-tazobactam and ciprofloxacin	Pipracillin-tazobactam and ciprofloxacin	-	Presumed cat exposure
11	Hamai et al. [28]	1999	49, male	No	P. multocida	Cat	Cefazolin (IP) and tobramycin (IP)	Cefazolin (IP) and tobramycin (IP)	-	Tubing punctured from cat bite
12	Chadah and Warady [29]	1999	18, male	No	P. multocida	Cat	-	-	-	Tubing punctured from cat bite
13	Langenhove et al. [30]	2000	22, female	No	P. multocida	Cat	Vancomycin (IP) and amikacin (IP)	Ciprofloxacin (PO)	-	Tubing punctured from cat bite
14	Martinez [31]	2000	-	-	-	-	-	-	-	-
15	Martinez [31]	2000	-	-	-	-	-	-	-	-
16	Martinez [31]	2000	-	-	-	-	-	-	-	-
17	Campos, et al. [3]	2000	8, male	No	P. pneumotropica	Hamster	Vancomycin (IP) and tobramycin (IP)	Vancomycin (IP) and tobramycin (IP)	Yes	Three pets in the home
18	Kanaan et al. [32]	2002	24, female	No	P. multocida	Cat and dog	Vancomycin (IV) and ciprofloxacin (PO)	Ciprofloxacin (PO)	-	Cat scratch to right hand
19	Sillery et al. [33]	2004	48, female	No	P. multocida	Cat	Cefazolin (IP) and gentamicin (IP)	Cefazolin (IP) and gentamicin (IP)	-	Cat licking hands of patient before and during dialysis
20	Cooke et al. [34]	2004	73, female	No	P. multocida	Cat	Vancomycin (IP) and gentamicin (IP)	Gentamicin (IP)	No	Cat exposure in house
21	Mat et al. [35]	2005	52, male	No	P. multocida	Cat	Cefazolin (IP) and amikacin (IP)	Cefazolin (IP)	-	Cat exposure in house
22	Malik et al. [36]	2005	58, male	No	P. multocida	Cat	Gentamicin	Gentamicin	-	Cat bite to patient
23	Malik et al. [36]	2005	21, female	No	P. multocida	Cat	Gentamicin, cefazolin and piperacillin-tazobactam	Gentamicin, cefazolin, piperacillin-tazobactam	-	Unknown
24	Olea et al. [37]	2006	46, female	No	P. multocida	Cat	Vancomycin (IP) and ceftazidime (IP)	Ceftazidime (IP)	-	Possible oral flora for patient, hematogenous spread
25	Anthony et al. [38]	2007	48, female	No	P. multocida	Dog	Gentamicin and Cefazolin	Gentamicin and cefazolin	-	Believed to have been from dog exposure, in house
26	Kazuko et al. [4]	2008	41, male	No	P. dagmatis	Cat	Cefazolin (IP) and tobramycin (IP)	Cefazolin (IP) and tobramycin (IP)	-	Cat ruptured infusion tube
27	Kazuko et al. [4]	2008	29, female	No	P. multocida	Cat	Cefazolin (IP) and tobramycin (IP)	Cefazolin (IP) and tobramycin (IP)	-	Unknown
28	Satomura et al. [39]	2009	58, male	No	P. multocida	Cat	Cefazolin (IP) and ceftazidime (IP)	Levofloxacin	-	Cat exposure, unknown of direct inoculation
29	Randon-Berrios et al. [40]	2010	38, male	No	P. multocida	Cat	Vancomycin (IP) and ceftazidime (IP)	Vancomycin and pipracillin-Ttazobactam (IV) -> Pipracillin-tazobactam (IV) -> ampicillin (IV)	-	Cat playing with dialysis tubing
30	Mugambi et al. [6]	2010	36, female	Yes, positive	P. multocida	Cat	Vancomycin (IP) and gentamicin (IP)	Ciprofloxacin (IV)	Yes	Cat exposure, unknown of direct inoculation
31	Nishina et al. [41]	2011	45, male	No	P. multocida	Cat	Vancomicin (IV) and ceftazidime (IP)	Levofloxacin (IV)	-	Cat punctured dialysis tubing
32	Weiss and Panesar [7]	2012	57, male	Yes, positive	P. multocida	Cat and dog	Vancomycin (IP) and ceftazidime (IP)	Vancomycin (IP) and ceftazidime (IP)	-	Cat exposure, unknown of direct inoculation
33	Sol et al. [42]	2013	7, female	No	P. multocida	Cat	Ceftazidime (IP) and cefazolin (IP)	Ampicillin (IP)	-	Cat exposure, unknown of direct inoculation
34	Al-Fifi et al. [43]	2013	49, male	No	P. multocida	Cat and dog	Vancomycin (IP) and tobramycin (IP)	Ceftazidime (IP)	-	Pet exposure, unknown of direct inoculation
35	Kim et al. [44]	2014	25, female	No	P. multocida	Cat	Cefazolin (IP) and gentamicin (IP)	Cefazolin (IP) and gentamicin (IP)	-	Cat exposure, unkown of direct inoculation
36	Dresselaars et al. [45]	2014	62, female	No	P. multocida	Cat	Cefalexin (PO) and cefalotin (IP)	Cotrimoxazole (IV) and cefalotin (IP)	-	Cat exposure, unknown of direct inoculation
37	Poliquin et al. [46]	2015	28, female	No	P. multocida	Cat	Cefazolin (IP) and tobramycin (IP)	Cefazolin (IP) and tobramycin (IP)	-	Similar cat exposure histories
38	Poliquin et al. [46]	2015	37, male	No	P. multocida	Cat	Cefazolin (IP) and tobramycin (IP)	Cefazolin (IP)	-	Cat bite to the dialysate tubing
39	Poliquin et al. [46]	2015	41, male	No	P. multocida	Cat	Cefazolin (IP) and tobramycin (IP)	Cefazolin (IP)	-	Cat bite to the dialysate tubing
40	Poliquin et al. [46]	2015	51, female	No	P. multocida	Cat	Cefazolin (IP) and tobramycin (IP)	Amoxicillin-clavulanic acid (PO)	-	Cat exposure, unknown of direct inoculation
41	Poliquin et al. [46]	2015	37, female	No	P. multocida	Cat	Cefazolin (IP) and ceftazidime (IP)	Ceftriaxone and amoxicillin (PO)	Yes	Cat exposure, unknown of direct inoculation
42	Poliquin et al. [46]	2015	59, female	No	P. multocida	Cat	Cefazolin (IP) and tobramycin (IP)	Ceftazidime (IP)	-	Cat exposure, unknown of direct inoculation
43	Poliquin et al. [46]	2015	69, female	No	P. multocida	Cat	Cefazolin (IP) and tobramycin (IP)	Cefazadime (IP) and amoxicillin-clavulinic acid (PO)	-	Cat bite to the dialysate tubing
44	Giron et al. [47]	2017	72, male	No	P. multocida	Cat	Ceftazidime (IP) and vancomycin (IP)	Ceftriaxone (IP)	-	Cat bite to the dialysate tubing
45	Tamura et al. [48]	2018	3, female	No	P. multocida	Cat	Cefazolin (IP) and piperacillin (IV)	Cefazolin (IV)	-	Cat scratch to the dialysis bag
46	Mirzai et al. [2]	2019	59, male	No	P. multocida	Cat	Vancomycin (IV) and ceftazidime (IV)	Ampicillin-sulbactam (IV) -> amoxicillin-clavulinic acid (PO)	-	Cat sleeping with him and inside house regularly
47	Adapa et al. [15]	2019	58, male	No	P. multocida	Cat	Vancomycin (IP) and ceftazidime (IP)	Ceftazidime (IP)	-	Cat exposure in house
48	Mastrapasqua et al. [49]^.^	2020	39, male	No	P. multocida	Cat	Vancomycin (IP) and gentamicin (IP)	Ceftriaxone (IP)	Yes	Cat exposure in house
49	Mu et al. [50]	2020	75, male	No	P. multocida	Cat	Levofloxacin (IV), ceftazidime (IP) and vancomycin (IP)	Meropenem (IV), ceftazidime (IP) and vancomycin (IP) -> cefoperazone-sulbactam (IV), amikacin (IP) and vancomycin (IP), imipenem-cilastatin IP -> ampicillin-sulbactam (IV) -> amoxicillin (PO)	No	Cat playing with tubing or contacted patient during continuous ambulatory peritoneal dialysis (CAPD)
50	Our case	2020	77, male	Yes, positive	P. multocida	Cat	Vancomycin (IV) and piperacillin-tazobactam (IV)	Piperacillin-tazobactam (IV) and cefepime (IP)	No	-

Based on clinical experience and in vitro data, *Pasteurella *infections are typically susceptible to penicillin, ampicillin, ampicillin-sulbactam, amoxicillin, amoxicillin-clavulanic acid, piperacillin-tazobactam or a carbapenem [8-11]. Conversely, the use of anti-staphylococcal penicillins such as oxacillin, nafcillin, or dicloxacillin should be avoided due to a lack of meaningful activity. β-lactamase production has been described in* P. multocida *strains with rare penicillin-resistant strains isolated in humans, primarily from the respiratory tract [8]. Cephalosporin activity increases with later generations as high minimum inhibitory concentrations can be seen with cephalexin, cefaclor, cefadroxil, or cefazolin; therefore, these agents should also be avoided [8,10]. Cefuroxime, ceftriaxone and ceftaroline, and ceftazidime demonstrate excellent in vitro activity and are considered good substitutes for penicillin, especially in penicillin-allergic patients [8-11]. Non-β-lactam antibiotic agents with in vitro activity used in clinical practice include tetracyclines, fluoroquinolones, and trimethoprim-sulfamethoxazole (TMP-SMX). Empiric treatment with aminoglycosides, clindamycin, or macrolides should typically be avoided [8,10].

This case presents a conundrum with the approach to antibiotic administration due to the presence of an intraperitoneal infection with concurrent bacteremia. On review of the most recent clinical practice guidelines, the International Society of Peritoneal Dialysis (ISPD) states as a 1B recommendation to use intraperitoneal antibiotics as first-line treatment unless the patient has concern for sepsis [12]. The ISPD does not make a specific practice recommendation for cases where the signs and symptoms of sepsis are present. In the case of our patient, he was found to have positive blood cultures, a viable source of infection, and end-organ dysfunction suggesting sepsis. In this scenario, the International Guidelines for Management of Sepsis and Septic Shock suggest as a 1C recommendation to utilize empiric intravenous antibiotics within one hour of diagnosis of sepsis [13]. Due to this, it was warranted to forgo the initial ISPD requirements and supplement his antibiotic therapy with an intravenous agent for broad-spectrum coverage. 

Duration of therapy varies by host factors, the severity of illness, and indication, with at least 14 days recommended for bacteremia [10,14]. Interestingly* P. multocida* peritonitis was previously successfully treated with a three-week course of intraperitoneal ceftazidime with the ability to salvage the dialysis catheter [15]. The use of intraperitoneal (IP) third cephalosporins, such as ceftazidime, opens up an intriguing option for susceptible *P. multocida *bacteremia and could potentially be considered for patients without reliable intravenous access based on pharmacokinetic data. The optimal dose is not clearly defined, but 1.5g IP Q24h has been suggested based on pharmacokinetic data [16,17]. The use of the loading dose as recommended by Cardone and colleagues for the indication of peritonitis (3 g IP once on day 1) requires clinical validation [17], but a systemic loading dose would be prudent in severe cases as therapeutic levels in the serum may be delayed due to diffusion and physiologic changes during the acute illness.

PD catheter removal is often indicated in cases of refractory or relapsing peritonitis. As this was our patient's first episode of peritonitis, the patient rapidly demonstrated initial clinical improvement; with negative screening blood and peritoneal fluid cultures, along with a rapid decrease in the peritoneal fluid WBC count, a decision was made not to remove his peritoneal dialysis catheter. The PD catheter in Weiss and Panesar's patient was also salvaged compared to Mugambi's case, in which the PD catheter was removed [6,7]. Both cases (our case and Weiss and Panesar's) achieved negative blood cultures within the first three days of antibiotic therapy.

As shown in Table 3 [6,7], among the reported patients with associated bacteremia, our patient presented with the highest overall burden of comorbidities based on the Charlson Comorbidity Index with and without adjustments for ESRD [51-53]. However, the patient reported by Mugambi et al. had a more severe presentation based on the quick Sequential Organ Failure Assessment (qSOFA) and the Shock Index scores. Our patient did suffer an acute worsening of his cardiac function, but as he had already cleared his infection prior to this deterioration, it's less likely that his outcome is mainly contributed to by his infection. It's more likely that the complex interaction between our patient's various comorbid conditions accentuated by the acute onset of an unusual infection contributed to his decompensation and transition to hospice care compared to the complete recovery of the other two reported cases with bacteremia. Blood cultures are often not done in the evaluation of patients with PD peritonitis in the absence of fever or suspected sepsis. Our case and that of Weiss and Paneser suggest that episodes of concomitant bacteremia are probably missed when blood cultures are omitted.

**Table 3:** Reported cases of P. multocida peritonitis complicated by bacteremia qSOFA - quick Sequential Organ Failure Assessment; ESRD - end-stage renal disease

	Charlson Comorbidity Index [51]	Charlson ESRD-adjusted Comorbidity Index [51]	qSOFA score [52]	Shock Index [52]	Outcome	Empiric antibiotics	Culture driven antibiotics	Time to negative blood culture	Peritoneal catheter removed?
Our case (2020)	7	6	2	1.1	Transition to hospice	Intravenous vancomycin and intravenous piperacillin-tazobactam	Intravenous piperacillin-tazobactam and intraperitoneal cefepime x 10 days	Hospital day 3	No
Weiss et al. (2012) [7]	2	0	Unable to calculate	Unable to calculate	Improved	Intraperitoneal vancomycin and ceftazidime	Intraperitoneal vancomycin and ceftazidime	Hospital day 2	No
Mugambi et al. (2010) [6]	3	3	3	1.4	Improved	Intravenous and intraperitoneal vancomycin and gentamicin	Intravenous ciprofloxacin x 14 days	Not reported	Yes

## Conclusions

Our case serves as a reminder to advocate for educating the peritoneal dialysis patients who have pets on the appropriate handling of PD equipment at home. Nevertheless, having pets at home can be beneficial for patients' emotional well-being. We want to echo Weiss and Panesar that there is a need for appropriate guidelines specifically tailored for PD patients who have pets at home. We believe that PD and having pets can co-exist, considering the right boundaries, hygiene, and knowledge. On the other hand, a discussion of risks and benefits should take place before initiation of PD in this special patient population as there are multiple reported cases of P. multocida PD associated peritonitis in patients who have cats at home with no direct exposure.
